# A case of choroidal neovascular membrane in 6-year-old boy with juvenile idiopathic arthritis

**DOI:** 10.1186/s12348-017-0136-1

**Published:** 2017-09-30

**Authors:** Parthopratim Dutta Majumder, Avirupa Ghose, Chetan Rao, Muna Bhende, Jyotirmay Biswas

**Affiliations:** 1 Department of Uvea, Sankara Nethralaya, 18 College Road, Chennai, 600006 India; 2 Department of Uvea, Aditya Birla Sankara Nethralaya, Kolkata, India; 3 Department of Vitreoretina, Sankara Nethralaya, Chennai, India

## Abstract

**Purpose:**

The purpose of this study is to report a case of choroidal neovascular membrane (CNVM) in a patient of juvenile idiopathic arthritis (JIA).

**Design:**

The design of this study is an interventional case report.

**Methods:**

A 6-year-old boy, a known patient of JIA, presented with a complaint of redness and ocular pain with mild diminution of vision in his left eye. Fundus examination revealed a CNVM with retinal thickening and subretinal fluid which was confirmed on OCT. Treatment with intravitreal injection of anti-VEGF (ranibizumab) and oral immunosuppressive showed resolution of the CNVM.

**Results:**

The result of this study is a successful treatment of CNVM with a single anti-VEGF injection and systemic immunosuppression.

**Conclusions:**

Although a rare complication, CNVM can occur in patients with JIA*.*

## Introduction

Choroidal neovascular membrane (CNVM) is a vision-robbing complication of uveitis. It is characterized by pathologic blood vessel growth from the choroid across Bruch’s membrane into the retina and often resulting in central vision loss. The incidence of CNVM due to uveitis has been reported to 2% and usually affects young individuals [1]. The prevalence of CNVM secondary to uveitis varies among different uveitic entities but is most commonly reported in patients with posterior uveitis and panuveitis [1–3]. CNVM in intermediate uveitis is relatively uncommon but has been reported in literature [4–6]. CNVM can occur as a result of any pathologic process that involves RPE and Bruch’s membrane [7].

Juvenile idiopathic arthritis (JIA) is the most common extraocular disease associated with uveitis in children. JIA-associated uveitis has plethora of clinical presentations and considered as a significant cause of ocular morbidity in children. Various complications have been reported to be associated with JIA: band keratopathy, cataract, glaucoma, ocular hypotony, and vision-robbing maculopathies like macular edema and epiretinal membrane. To the best of our knowledge, CNVM secondary to JIA has not been yet reported in literature. We, in hitherto, report a case of CNVM in a patient of JIA.

## Case report

**Fig. 1:**
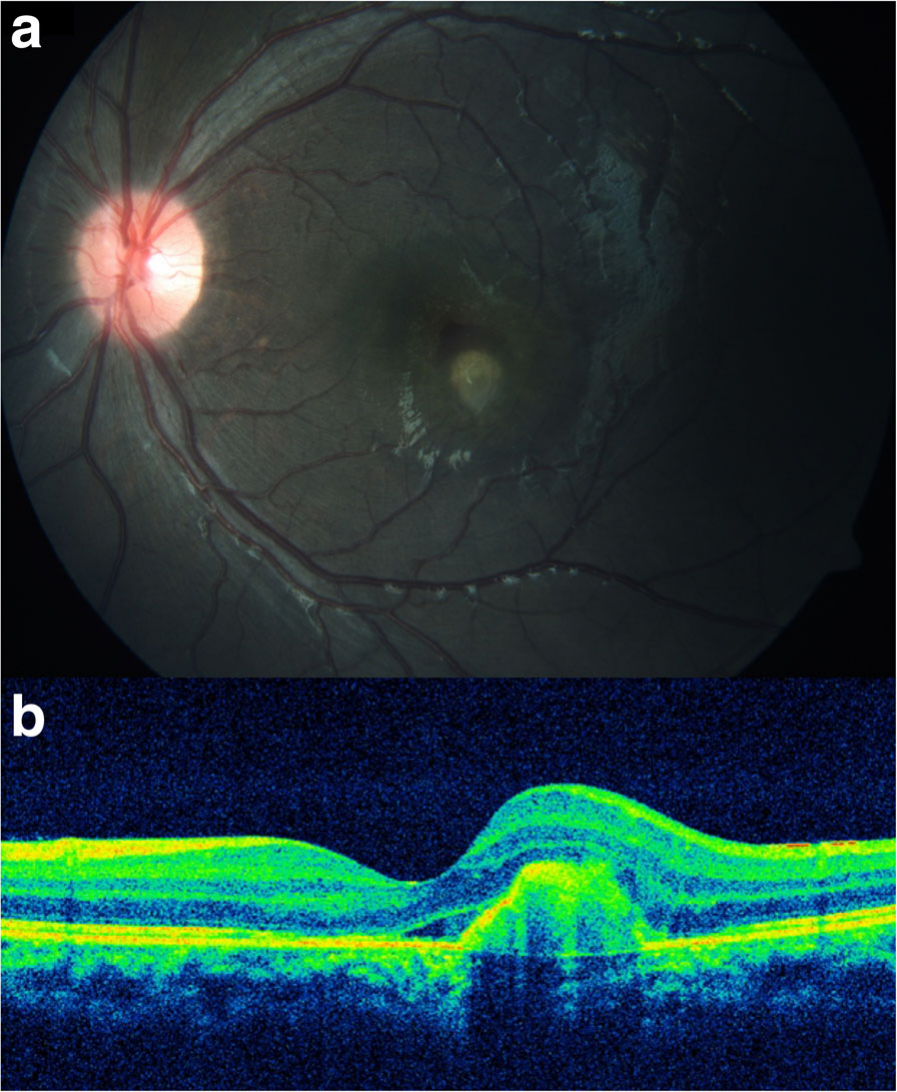
**a** Color fundus photograph of the left eye on presentation. **b** Optical coherence tomography through the lesion showing the choroidal neovascular membrane, retinal thickening, and subretinal fluid

**Fig. 2:**
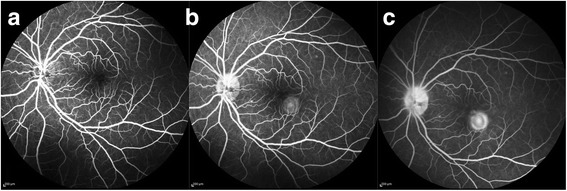
Fundus fluorescein angiography of the *left* eye showing **a** no obvious leakage in the early phase, **b** faint hyperfluorescence in the mid phase, and **c** intense hyperfluorescence with leakage in the late phase

**Fig. 3:**
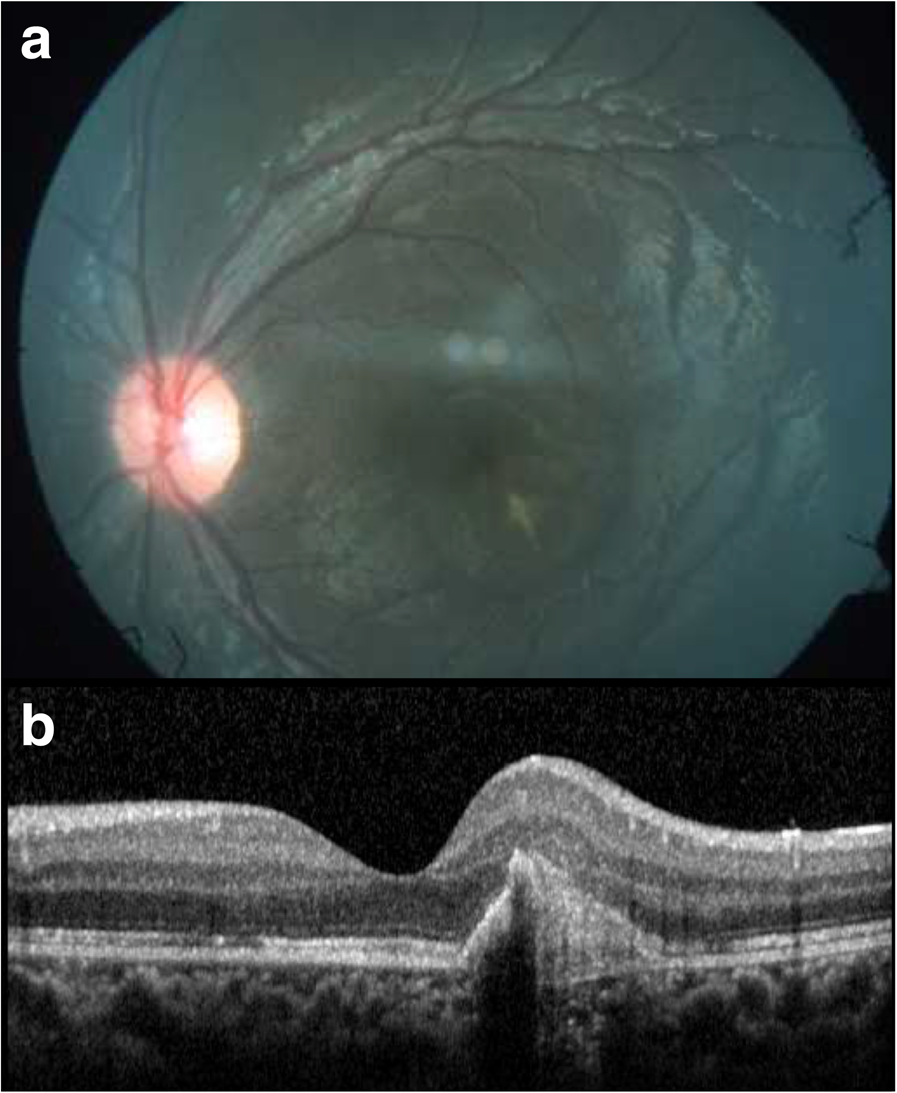
**a** Color fundus photograph of the *left* eye at 1-month follow-up. **b** Optical coherence tomography showing the scarred choroidal neovascular membrane and resolution of subretinal fluid

A 6-year-old emmetropic boy, a known patient of JIA, presented to our outpatient department with a complaint of redness and ocular pain with mild diminution of vision in his left eye. He was started on oral methotrexate on account of recurrent attacks of anterior uveitis 3 months back, and at the time of presentation, he was on oral methotrexate 7.5 mg/week. He was extensively investigated to rule out other possible causes of intraocular inflammation including infectious etiology. His best-corrected visual acuity (BCVA) on presentation was 6/7.5 in the left eye. Ocular examination revealed a decrease in best-corrected visual acuity of 6/9 in the left eye. Slit-lamp examination of the right eye showed quiet anterior chamber and few cells in anterior vitreous. Fundus examination of the right eye was normal. Slit-lamp examination of the left eye showed early band-shaped keratopathy, occasional cells (0.5+) in anterior chamber and cells in anterior vitreous. Fundus examination of the left eye showed a yellowish lesion with overlying subretinal fluid just inferior to fovea (Fig. 1a). Fundus fluorescein angiography of the left eye showed no obvious leakage in the early phase, faint hyperfluorescence in the mid phase, and intense hyperfluorescence with leakage in the late phase (Fig. 2a–c). Optical coherence tomography of the left eye showed a CNVM with retinal thickening and subretinal fluid (Fig. 1b). A diagnosis of inflammatory CNVM was made. Dose of oral methotrexate was hiked up to 10 mg/week, and intravitreal injection of anti-VEGF (ranibizumab) was administered in the left eye. Patient was examined again after a month. His BCVA improved to 6/6, and slit-lamp examination of his left eye showed a quiet AC and few old, pigmented cells in anterior vitreous. Fundus examination of the left eye showed healed CNVM and resolution of subretinal fluid, which was confirmed by optical coherence tomography (Fig. 3a, b). Patient was maintained on methotrexate 10 mg weekly. Patient is under a regular follow-up with us for last 9 months. There has been no recurrence in uveitis or CNVM till date.

## Discussion

Literature on posterior segment involvement in JIA is relatively rare [8–11]. Macular involvement in JIA might be much more common than has been reported in literature. In a retrospective review of 67 patients with JIA, 37.1% had posterior complications, 13.8% had macular edema, 12% papillitis, 6.8% epiretinal membrane, 2.5% retinal vasculitis, and 1.7% retinal detachment [11]. In a cross-sectional prospective study in 62 eyes of 38 patients with JIA-associated uveitis, maculopathy was noted in 82% of the eyes [9]. This study highlights the importance of optical coherence tomography, which can detect subtle macular changes that are not identifiable on biomicroscopy.

Our findings of CNVM in a child with JIA may be explained with the concept that CNVM is driven at least in part by the intraocular inflammation. CNVM is relatively uncommon among patients with anterior and intermediate uveitis but not rare [6, 12]. Breakdown of blood-retinal barrier and the role of several inflammatory and vasoactive peptides have been implicated as the cause of macular edema in JIA [13]. Our case clearly demonstrated OCT and angiographic evidence of CNVM in a patient of JIA in the absence of other identifiable causes. Based on our finding, we conclude that CNVM is a rare complication of JIA and usually have a favorable outcome if diagnosed early and treated with anti-VEGF and systemic immunosuppression.

## Conclusion

To the best of our knowledge, this is the first reporting of CNVM in a patient with JIA. Though occurrence of CNMV can be multifactorial, the absence of other factors like myopia and history of trauma in a child with on-going intraocular inflammation helped us arrive at this conclusion.

## References

[1] Baxter SL, Pistilli M, Pujari SS (2013). Risk of choroidal neovascularization among the uveitides. Am J Ophthalmol.

[2] Kuo IC, Cunningham ET (2000). Ocular neovascularization in patients with uveitis. Int Ophthalmol Clin.

[3] Moorthy RS, Chong LP, Smith RE, Rao NA (1993). Subretinal neovascular membranes in Vogt-Koyanagi-Harada syndrome. Am J Ophthalmol.

[4] Arkfeld DF, Brockhurst RJ (1985). Peripapillary subretinal neovascularization in peripheral uveitis. Retina Phila Pa.

[5] Garcia CA de A, Segundo P de S, Garcia Filho CA de A, Garcia ACM de A. Intermediate uveitis complicated by choroidal granuloma following subretinal neovascular membrane: case reports. Arq Bras Oftalmol 2008;71(6):890-893 10.1590/s0004-27492008000600026 19169529

[6] Mehta S, Hariharan L, Ho AC, Kempen JH (2013). Peripapillary choroidal neovascularization in pars planitis. J Ophthalmic Inflamm Infect..

[7] Campa C, Costagliola C, Incorvaia C et al (2010) Inflammatory mediators and angiogenic factors in choroidal neovascularization: pathogenetic interactions and therapeutic implications. Mediat Inflamm 2010. doi:10.1155/2010/546826 10.1155/2010/546826 PMC2943126 20871825

[8] Chen CS, Roberton D, Hammerton ME (2004). Juvenile arthritis-associated uveitis: visual outcomes and prognosis. Can J Ophthalmol J Can Ophtalmol.

[9] Ducos de Lahitte G, Terrada C, Tran THC (2008). Maculopathy in uveitis of juvenile idiopathic arthritis: an optical coherence tomography study. Br J Ophthalmol.

[10] Paroli MP, Speranza S, Marino M, Pirraglia MP, Pivetti-Pezzi P (2003). Prognosis of juvenile rheumatoid arthritis-associated uveitis. Eur J Ophthalmol.

[11] Paroli MP, Spinucci G, Fabiani C, Pivetti-Pezzi P (2010). Retinal complications of juvenile idiopathic arthritis-related uveitis: a microperimetry and optical coherence tomography study. Ocul Immunol Inflamm.

[12] Heymann HB, Colon D, Gill MK (2015) Choroidal neovascularization secondary to tubulointerstitial nephritis and uveitis syndrome (TINU) in an adult patient. J Ophthalmic Inflamm Infect 5. doi:10.1186/s12348-015-0059-7 10.1186/s12348-015-0059-7 PMC4596145 26446047

[13] Paroli MP, Fabiani C, Spinucci G, Abicca I, Sapia A, Spadea L (2013) Severe macular edema in patients with juvenile idiopathic arthritis-related uveitis. Case Rep Ophthalmol Med 2013. doi:10.1155/2013/803989 10.1155/2013/803989 PMC3760270 24024057

